# A Review on the Prevalence and Treatment of Antibiotic Resistance Genes in Hospital Wastewater

**DOI:** 10.3390/toxics13040263

**Published:** 2025-03-31

**Authors:** Lihua Lan, Yixin Wang, Yuxin Chen, Ting Wang, Jin Zhang, Biqin Tan

**Affiliations:** 1 Key Laboratory of Recycling and Eco-Treatment of Waste Biomass of Zhejiang Province, School of Environment and Natural Resources, Zhejiang University of Science and Technology, Hangzhou 310023, China; lanlihua_011@zju.edu.cn (L.L.); wangyixin0605@163.com (Y.W.); chenyuxin_777@163.com (Y.C.); fywangting@163.com (T.W.); 2 Key Laboratory of Clinical Cancer Pharmacology and Toxicology Research of Zhejiang Province, Department of Pharmacy, Affiliated Hangzhou First People’s Hospital, School of Medicine, Westlake University, Hangzhou 310006, China

**Keywords:** antimicrobial resistance, antibiotic resistance genes, hospital, wastewater, COVID-19

## Abstract

Antibiotic resistance is a global environmental and health threat. Approximately 4.95 million deaths were associated with antibiotic resistance in 2019, including 1.27 million deaths that were directly attributable to bacterial antimicrobial resistance. Hospital wastewater is one of the key sources for the spread of clinically relevant antibiotic resistance genes (ARGs) into the environment. Understanding the current situation of ARGs in hospital wastewater is of great significance. Here, we review the prevalence of ARGs and antibiotic-resistant bacteria (ARB) in hospital wastewater and wastewater from other places and the treatment methods used. We further discuss the intersection between ARGs and COVID-19 during the pandemic. This review highlights the issues associated with the dissemination of critical ARGs from hospital wastewater into the environment. It is imperative to implement more effective processes for hospital wastewater treatment to eliminate ARGs, particularly during the current long COVID-19 period.

## 1. Introduction

Antibiotic resistance (AR) is a growing threat to public health worldwide. The WHO formulated the Call to Action on Antimicrobial Resistance (AMR)-2021 on 29 April 2021. China is one of the largest producers and consumers of antimicrobial drugs worldwide [1]. In recent decades, our country has established CARSS, CFDSS, and the Center for Antibacterial Surveillance. The International Nosocomial Infection Control Consortium (INICC) reported that 46% of hospital-acquired infections are due to *Enterobacteriaceae* and 27% are due to *Pseudomonas* spp., followed by 6% *Acinetobacter* spp., 8% *Candida* spp., and 3% *Staphylococcus* spp. [2]. The extensive application of antibiotics for the prevention and treatment of human infections has resulted in the emergence of antibiotic-resistant bacteria (ARB) and antibiotic resistance genes (ARGs) within hospital settings.

Carbapenem and some other important antibiotics are often prescribed in hospitals by doctors, which is different from other antibiotics, such as sulfanilamide and flufenicol, which are commonly used in farming. Therefore, ARGs’ characteristics in hospital settings are unique. The prevalence of carbapenem-resistant *Klebsiella pneumoniae* (CR-KPN) in China rose from 6.4% in 2014 to 10.8% in 2023. The prevalence of resistance to the third-generation cephalosporin among *Escherichia coli* (CTX/CRO-R-ECO) and *Klebsiella pneumoniae* (CTX/CRO-R-KPN) in 2023 was observed to be 48.9% and 36.9%, respectively, indicating persistently high detection rates. The incidence of methicillin-resistant *Staphylococcus aureus* (MRSA) has demonstrated a steady decline in recent years, decreasing from 37.1% in 2012 to 29.1% in 2023 according to the 2023 Surveillance Report of Bacterial Resistance in China. As the primary source for ARB and ARG development, hospitals have been traditionally regarded as hotspots [3,4,5,6].

Hospitals are key sources of antibiotic resistance in the environment [7,8]. A substantial body of research has reported that hospital-related ARB and ARGs are present in the sewage environment [9,10,11,12,13,14]. ARB and ARGs are commonly identified in hospital wastewater (HWW), wastewater treatment plants (WWTPs), and subsequently in WWTP effluent [15,16,17,18]. As a result, the problem of AR is related not only to the inappropriate and excessive use of antibiotics in the clinic but also to their discharge into sewage, which might contribute to its prevalence in ecosystems. Tripartite (FAO, OIE, and WHO) and UNEP support OHHLEP’s definition of “One Health”, which means that the AR is a problem that requires cooperation [19,20]. The dissemination of ARB and ARGs from HWW in the environment impairs water ecology, ultimately harming human health. Understanding the prevalence of antibiotic resistance and the treatment of ARB and ARGs in HWW are critical for human health.

The purpose of this review is to compare the prevalence of critical ARGs and ARB in HWW with that in other environments. We emphasize the necessity for hospitals to strengthen pretreatment protocols prior to discharging wastewater to WWTPs. Furthermore, such measures can mitigate the environmental propagation of antibiotic resistance, particularly during public health emergencies when antibiotics are extensively utilized, as exemplified by the recent pandemic.

## 2. The Prevalence of ARB and ARGs in HWW

HWW represents a minor fraction of the overall volume of wastewater processed in wastewater treatment plants (WWTPs), generally comprising less than 2% of the total [21]. However, much larger quantities of antibiotics are used for farm animals, community settings, and retirement homes. But it is important to note that critical ARB and ARGs have been more frequently identified in HWW. Comparing ARBs and ARGs in hospitals with those in other resources is important for investigating the role of HWW in the dissemination of ARBs and ARGs in aquatic environments.

Researchers have conducted various comparative analyses of ARB and ARGs in wastewater samples from HWW and other sources [3,22,23]. Six types of ARGs were selected for analysis, including carbapenem resistance genes (such as *blaNDM-1* and *blaKPC*), the methicillin resistance gene from staphylococci (*mecA*), the vancomycin resistance gene from *enterococci* (*vanA*), resistance genes to extended-spectrum beta-lactamases (*ESBLs*), the colistin resistance gene of Gram-negative bacteria (*mcr-1*), and sulfonamides. A few studies have investigated resistance genes to tetracyclines, quinolones, glycopeptides, chloramphenicol, and mobile genetic elements (MGEs).

### 2.1. Methods Used for Analyzing ARGs and ARB

Let us first review the detection methods of ARB and ARGs. For decades, the culture-based method has mainly been used to identify resistant microorganisms. Selective media such as antibiotic-supplemented agar (e.g., MacConkey agar with cefotaxime for ESBL-producing *Enterobacteriaceae*) are used for isolating ARBs, followed by antibiotic susceptibility testing using disk diffusion or microdilution assays (according to the CLSI/EUCAST guidelines) to determine resistance profiles against clinically relevant antibiotics. The purification sensitivity test obviously has limitations as only a few environmental bacteria can be cultured under laboratory conditions.

Molecular methods were employed to detect ARB without the need for culturing. Conventional PCR testing confirmed gene presence/absence, while quantitative PCR (qPCR) methods were used to quantify ARGs using species-specific primers designed for known ARGs. Digital PCR (dPCR) testing is used for the absolute quantification of low-abundance ARBs with improved precision in complex matrices.

Metagenomics enables the high-throughput identification of ARGs in complex microbial communities without culturing by sequencing total DNA from clinical, wastewater, or environmental samples, followed by bioinformatic analysis (e.g., alignment to ARG databases like CARD or ResFinder). Researchers are able to characterize the diversity, abundance, and potential horizontal transfer of resistance genes. This culture-independent approach encompasses both known and novel antibiotic resistance genes (ARGs), providing valuable insights into the dynamics of the resistome across various ecosystems and informing public health interventions [24]. However, this approach demands costly testing equipment, high testing costs, and significant expertise for the analysis of results, which many hospitals and research institutions lack.

In this review, the researchers employed the aforementioned methodologies to identify ARBs and perform the quantitative analysis of ARGs.

### 2.2. Carbapenem Resistance

Carbapenem-resistant Enterobacteriales (CRE) is identified as one of the most high-priority pathogens by the World Health Organization (WHO) due to its capacity to induce life-threatening infections that are increasingly challenging to manage with antibiotics. The primary mechanism of resistance in CRE is the production of carbapenemase. Several major types of carbapenemase genes have been documented in hospitals and other places. Compared with those in other environments, the levels of carbapenem-resistant bacteria and carbapenemase genes in HWW were significantly higher in almost all studies; statistical significance was evaluated in part of these studies (Table 1). Only two studies demonstrated a slightly higher prevalence in other environments. Heike Muller et al. [25] detected *blaIMI* and *blaVIM* in a rural system, whereas *blaIMI* was not detected in a clinical/urban system. Meir-Gruber et al. [26] observed a similar prevalence of *blaNDM-1-* and *blaKPC*-positive samples in hospital and community sewage. The authors noted that it remains uncertain whether the presence of pan-resistant bacteria in the community is attributable to person-to-person transmission with hospitals serving as a significant reservoir, or whether other environmental factors (food or water consumption, etc.) play a role. These findings underscore the critical role of carbapenem-resistant bacteria and carbapenemase genes in HWW as sources of antibiotic resistance dissemination in the environment.

### 2.3. MRSA

MRSA is another important clinically relevant ARB detected in HWW, and the presence of MRSA and its resistance gene *mecA* in other environments was undetectable in this comparative study (Table 2). Schwartz identified the presence of *mecA* in hospital wastewater biofilms, with no detection in any other compartments [36]. Meir-Gruber investigated the prevalence of MRSA in hospital versus community sewage and found that none of the districts exhibited MRSA-positive samples within the community [26]. However, Johannes Alexander detected mecA in hospital sewers (3.26 × 10^−7^/16S copy number) and retirement home sewers (2.55 × 10^−7^~8.19 × 10^−5^/16S copy number) [27]. The author suggested the presence of a nursing home unit within retirement homes may account for the observed ARG patterns, which are analogous to those found in hospital sewers. In addition, they detected the presence of *mecA* sporadically in housing sewers. These results suggest that retirement homes should also be considered when detecting the distribution of clinically relevant MRSA in addition to hospitals.

### 2.4. VRE

VRE (vancomycin-resistant *Enterococci*) is among the most worrisome pathogens in hospitals around the world. Studies have focused on the expression of *vanA* in VRE^+^ samples and the presence of *vanA* in hospital wastewater and other places [27]. More than half of the studies reported higher frequencies of VRE and *vanA* in hospital wastewater. *VanA* was not detected in retirement homes or housing sewers. A greater number of vancomycin ARGs were also detected in hospital wastewater than in community wastewater in Northwest Spain [21]. Caplin observed an inverse trend, attributing the relatively low prevalence of VRE in hospital wastewater to the heterogeneous nature of the samples, which commonly included disinfectants and detergents. Similarly, Blanch reported a widespread VRE prevalence in community wastewater compared to that in hospital settings. This discrepancy may be due to the fact that bacteria from HWW originate from a specific subset of the human population, and the hospital environment selectively favors certain bacterial strains. In conclusion, hospitals contribute a higher abundance of VRE, as illustrated in Table 3.

### 2.5. ESBL

The majority of studies indicated that the prevalence of *E. coli* possessing ESBLs was greater in hospital settings compared to other environments (Table 4). In addition to the slightly lower number of copies of *blaTEM* and *blaSHV* in hospital wastewater, the hospital wastewater had higher concentrations of ESBL-(*blaCTX*, *blaTEM*, *blaSHV*, and *blaOXA*) ARGs, implying that in-hospital antibiotic use should be more strictly controlled in regards to ESBL.

### 2.6. Other Resistance Mechanisms

In addition to resistance to last-resort antibiotics, which was previously described, multiple mobile colistin resistance (*mcr*) genes, as well as *optrA* and *cfr* genes—which can confer resistance to linezolid—were also identified (Table 5). Researchers indicated that the majority of these ARBs or ARGs were more prevalent in hospital settings compared to other environments. However, the prevalence of *mcr* genes was higher in municipal wastewater than in HWW. The authors attributed this observation to the significant contribution of Aeromonas from biofilms as the wastewater travels through the sewer system. Regarding *sul4*, *gar*, and *cmlA1* genes, their prevalence rates were comparable between hospital and non-hospital environments.

## 3. ARG Treatment in HWW

As described above, ARB and ARGs are generally more abundant in hospital effluent than in other places, although the prevalence of clinically relevant MRSA in retirement homes is higher. The current situation in which ARGs are treated in hospital wastewater is a matter of significant concern. In some countries, HWW discharges into wastewater treatment plants (WWTPs) without pretreatment, and they are co-treated with urban wastewater [52]. Discharging hospital sewage directly into municipal wastewater treatment plants without prior treatment can potentially lower operational costs for hospitals.

Here, we categorized hospital wastewater treatment methods into two types: pretreatment before entering the urban WWTPs; and direct discharge into the urban wastewater network without prior treatment (Table 6). From a technical perspective, contemporary sewage treatment technologies are primarily categorized into biological methods, such as the activated sludge process and membrane bioreactors, and chemical methods, including various advanced oxidation processes [53]. Biological treatment uses microorganisms to break down organic matter in wastewater. The biological methods offer advantages such as being environmentally sustainable, cost-effective, and efficient in organic matter removal. However, they are characterized by slower processing times and the extensive area of the wastewater treatment facility and may exhibit a reduced efficacy against ARGs. Chemical treatment involves using chemicals to remove contaminants. The chemical approach presents several advantages, such as rapid treatment, broad applicability, and consistent performance. However, it also presents certain disadvantages, including high operational costs, the production of toxic byproducts, and potential environmental impacts.

Most WWTPs use a combination of conventional activated sludge (CAS) and disinfection processes, most of which use chlorination and some of which use ultraviolet (UV) disinfection or ozonation. In some studies, these processes effectively reduce ARGs after hospital sewage treatment [21,29,31,36,54,55,56,57,58]. Min Cai et al. demonstrated that the chlorine dioxide disinfection process significantly reduced ARGs, although following treatment, negative removal rates of *blaTEM-1* (−52.93%) and *tetB* (−15.37%) were observed. The absolute concentrations of *intI1* (75.95%) and *16S rRNA* (58.59%) genes significantly decreased following treatment, demonstrating the process’s efficacy in mitigating the risk of vertical propagation and the horizontal transfer of ARGs [59]. Lin Zhu et al. observed that chlorine disinfection successfully reduced both the absolute (copies/L) and relative abundances (copies/cell) of most last-resort ARGs (LARGs) [60]. However, the wastewater ecosystem remains a critical reservoir for LARGs, facilitating the colocalization of *blaNDM*, *tet(X)*, and *mcr* on the same plasmid, which poses a substantial threat to clinical and public health. Other studies have shown a small decline in ARGs following the treatment of hospital sewage. In a study conducted by Xueli Ma et al., the average total abundance of ARGs was measured in wastewater from both ophthalmic and general hospital wastewater. The findings demonstrated that WWTPs resulted in a slight decrease in the prevalence of ARGs [61]. Similarly, Sofia Svebrant and Alisha Akya reported that traditional wastewater treatment methods may not completely eradicate resistance genes [62,63].

In addition to a poor removal efficiency, the traditional process has corresponding drawbacks [64,65]. Chlorination and ozonation, while commonly employed in water reuse processes, have the potential to generate carcinogenic byproducts, with ozonation also being associated with high operational costs. UV disinfection offers several advantages, including a compact design, the absence of chemical residues, and efficacy in reducing ARGs. Nevertheless, it presents challenges such as the potential for bacterial regrowth and the photoreactivation of pathogenic microorganisms. Consequently, there is an urgent imperative to develop and implement more effective and cost-efficient technologies for wastewater treatment systems. Currently, various innovative technologies, including photocatalytic oxidation technology, Fenton oxidation technology, electrochemical oxidation technology, supercritical water oxidation technology, and other advanced oxidation processes, have been rapidly developed and implemented to enhance the capacity of sewage treatment plants to reduce the levels of ARB and ARGs. Among these, the membrane separation process has demonstrated the most promising results in diminishing ARGs, despite its high cost. On-site treatments utilizing advanced technologies, including ozonation, membrane bioreactor (MBR) treatment, UV treatment, and granulated activated carbon, have shown positive outcomes in the elimination of ARGs, with MBR treatment being the most effective for ARG reduction and ozonation for antibiotic reduction [66]. Zheng et al. have introduced a novel approach, E-peroxone-SBR, which offers an effective method for mitigating the production of ARGs [67].

However, despite these efforts, there are still challenges to overcome. The complexity and diversity of hospital sewage compositions make it difficult to achieve the complete and consistent removal of resistance genes. Moreover, the cost and practicality of implementing new treatment technologies on a large scale also pose significant obstacles. Furthermore, large hospitals are generally situated in densely populated urban areas, where limited land availability constrains the development of advanced on-site wastewater treatment facilities.

In conclusion, while progress has been made in understanding and addressing the issue of ARGs in hospital sewage, much work remains to be done to develop efficient, cost-effective, and sustainable treatment strategies to minimize the potential risks associated with these genes.

**Table 6:** Comparison of ARGs between before and after wastewater treatment.

Study	Samples	Strategy	ARG	Before Treatment	After Treatment
[60]	Hospital in China	Chlorination	the relative abundance of LARGs (*blaNDM*, *mcr*, *tet(X)*)	influent > effluent (*p* < 0.05)	
[61]	Eye hospital in China	Activated sludge wastewater treatment	The average total abundance of ARGs	1.614 ± 0.177	1.240 ± 0.237
	General hospital in China	Direct chlorination disinfection wastewater treatment		1.883 ± 0.451	1.278 ± 0.048
[59]	Hospital in China	Chlorine dioxide disinfection process	The removal efficiencies of various genes (%)	*sul1*:55.75%; *sul2*:87.62%; *sul3*:93.51%	
				*aadA*:79.33%; *aac(6′)-Ib*: 90.21%;	
				*ereA*:70.86%; *ermB*:85.76%	
				*blaNDM-1*:89.75%; *penA*: 69.55%	
				*tetM* eliminated after treatment	
				*intI1*:75.95%; *16S rRNA*:58.59%	
				*tetB*:−15.37%; *blaTEM-1*:−52.93%	
[58]	Hospital in China	WWTP	log removal of ARGs (=log(C_untreated_/C_treated_)	−0.85~2.71	
[54]	Hospital in Iran	Ozonation	The removal efficiencies of *P. aeruginosa* concentration 10^8^ CFU/mL	1 × 10^−1^ (TOD1 = 11 mg/L) 1 × 10^−4^ (TOD4 = 45 mg/L)	
[63]	Hospital in Sweden	Ozonation	CFU/mL of *ESBL*-producing Enterobacteriaceae	14,500 ± 4910	12,300 ± 1160
[29]	Hospital in France	WWTP	The normalized cumulative abundance of all gene classes	Hospital WWTP influent > WWTP effluent (78 and 5 times, *p* < 0.003)	
[57]	Hospital in Irea	WWTP	Copies/genome equivalent *mefA*	0.2	0.03
			Copies/genome equivalent *mel*	0.17	0.03
[62]	Hospital in Iran (*MSSA*, *n* = 10)	WWTP	*mec A* (*p* = 1)	1(10%)	1(10%)
			*vanA* (*p* = 0.305)	1(10%)	0
			*vanB*	0	0
			*vanC* (*p* = 0.305)	1(10%)	0
			*aacA-D* (*p* = 0.051)	1(10%)	5(50%)
			*tetK* (*p* = 1)	3(30%)	3(30%)
			*tetM* (*p* = 0.160)	5(50%)	8(80%)
	Hospital in Iran (*MRSA*, *n* = 20)	WWTP	*mec A* (*p* = 1)	20(100%)	20(100%)
			*vanA*	0	0
			*vanB*	0	0
			*vanC* (*p* = 0.429)	3(15%)	5(25%)
			*aacA-D* (*p* = 0.072)	20(100%)	17(85%)
			*tetK* (*p* = 0.001)	19(95%)	9(45%)
			*tetM* (*p* = 0.185)	9(45%)	5(25%)
[66]	HWW (location 1), two sampling rounds in the Netherlands	Ozonation, membrane bioreactor treatment (MBR), UV treatment, and granulated activated carbon (GAC)	Log10-fold gene reduction	Pharmafilter *	
			*blaKPC*	>1.7 ^1,3^	
			*blaSHV*	>3.1 ^1,3^	
			*blaOXA*	>3.6 ^1,3^	
			*qnrS*	>0.9 ^3^	
			*mecA*	^2^	
			*ermB*	>4.4 ^1,3^	
			*tetM*	>3.1 ^1,3^	
			*tetB*	>3.1 ^1,3^	
			*vanA*	>2.4 ^3^	
			*int1*	0.5 ± 0.9 ^1^	
			*ermF*	1.7 ± 0.8	
			*aph(III)a*	>3.8 ^1,3^	
			*sul1*	1.7 ± 0.4 ^1^	
[31]	Hospital in Sweden (together with household)	WWTP	Number of ARGs	44	33
[21]	Hospitals and community in Spain	WWTP	vancomycin ARG/genome	0.037 ± 0.006	<0.001
[67]	Hospital in China	E-peroxone-SBR	gene reduction of *qnrS*, *qnrA*, *qepA aac(6′)-Ib-cr aac(6′)-Ib-cr*	*p* < 0.05	
[55]	Urban hospital in Vietnam	WWTP	*blaCTX-M*	14 (52%)	2 (17%)
			*blaTEM*	27 (100%)	10 (83%)
			*qepA*	12 (86%)	7 (88%)
[35]	Hospital in Spain	WWTP	Absolute concentration [log (ARG copies/mL)] *blaTEM*, *ermB*, *qnrS*, *sul1*, *tetM*	influent > effluent (*p* < 0.05)	
[51]	Hospital in Iran	Chlorination (HW3: Chlorination + UV)	*ctx-m-32* presence/absence	+	-

^1^ reduced to <LOD, ^2^ non-quantifiable reduction from <LOQ to <LOD, ^3^ significantly reduced, Pharmafilter reduction significantly higher than UWWTP reduction, * Mean and SD for log10-fold gene reduction in Pharmafilter.

## 4. COVID-19 and Antimicrobial Resistance

Coronavirus disease 2019 (COVID-19) is a viral disease that often induces bacterial pneumonia, which requires antibiotic treatment. A study in “The Lancet” from Wuhan Pulmonary Hospital, China revealed that 50% of COVID-19 patients had secondary bacterial infections [68]. On the basis of this background, antibiotics are widely used in clinical trials on a global scale, increasing the inevitable ARB and ARG burden.

Only 8% of COVID-19 patients acquired bacterial/fungal superinfections that required antibiotic treatment, according to the Infectious Diseases Society of America (IDSA) reports [69]. Indeed, 72% of COVID-19 patients received antibiotic treatment despite a paucity of evidence of bacterial superinfection [70,71,72]. The antibiotic administration rate ranges from 33% to 95% for COVID-19 patients worldwide [68,73,74].

At the beginning of the epidemic, on the basis of limited evidence at that time, it was challenging for clinicians to confirm whether patients’ symptoms were related to bacterial infection. The standard guidelines also recommend antibacterial drugs, such as benzylpenicillin, metronidazole, moxifloxacin, and ciprofloxacin, as first-line drugs. Certainly, with a deeper understanding of the epidemic, the guidelines are constantly updated with stronger evidence for drug use.

During the COVID-19 pandemic, AMR has been documented among COVID-19 patients in hospital settings [75,76,77]. Numerous countries have reported the presence of AMR in hospitalized COVID-19 patients. Specifically, NDM-producing Enterobacter cloacae and vanB clones have been identified in critically ill COVID-19 patients in the United States and Germany [78,79]. The AMR strains isolated from these patients exhibited resistance to third-generation cephalosporins and carbapenems, with resistance rates ranging from 64% to 69% [80]. Additionally, several studies have reported the ineffectiveness of antimicrobial therapy in treating Aspergillus infections [81,82,83].

A. Johar et al. investigated the ARG profiles of pathogens present in HWW from both COVID-19 isolation hospitals and non-COVID-19 healthcare facilities during the pandemic. The analysis identified a total of 61 ARGs across the four hospitals examined. As anticipated, the COVID-19 isolation hospitals exhibited the highest prevalence of ARGs, accounting for 88.5% of the total, whereas the non-COVID-19 facilities showed the lowest prevalence [84]. Furthermore, research by Changzhi Wang et al. demonstrated a positive correlation over time between ARGs resistant to tetracyclines, sulfonamides, and macrolides in Hospital A, which treated COVID-19 patients, but not in Hospital B, which treated non-COVID-19 patients [85]. Over time, and with an increase in the number of COVID-19 patients, there was a significant positive selection pressure for carbapenem-resistant ARGs particularly associated with mobile genetic elements [86]. This observation underscores the potential dissemination of ARGs resistant to last-resort antibiotics through HGT (horizontal gene transfer). Consequently, it is crucial to enforce stewardship for appropriate antibiotic use within hospitals and the community during the pandemic. Additionally, ensuring the efficient treatment of both hospital and municipal wastewater is essential. Overall, this dual approach can help to mitigate unintentional contributions to the threat of AMR during the COVID-19 pandemic.

The rise in AMR following the COVID-19 pandemic is incontrovertible. It is hypothesized that there will be a significant shortage of effective antibiotics as the incidence of multidrug-resistant and extensively drug-resistant cases continues to rise [87,88,89]. The global disruption of healthcare services during the pandemic has adversely affected the treatment of other critical diseases, potentially leading to the emergence of new antibiotic resistance patterns. Furthermore, the escalation of AMR in environmental contexts contributes to the introduction of novel resistance genes.

## 5. Discussion

The global dissemination of antibiotic resistance genes (ARGs) and antibiotic-resistant bacteria (ARB) in hospital wastewater (HWW) constitutes a critical intersection of clinical practice, environmental contamination, and public health concerns. This review synthesizes substantial evidence indicating that HWW contains significantly elevated levels of clinically relevant ARGs—particularly those encoding carbapenemases (e.g., *blaNDM* and *blaKPC*), extended-spectrum β-lactamases (ESBLs), and methicillin resistance determinants (*mecA*)—in comparison to municipal or community wastewater. These disparities are likely attributable to the extensive use of broad-spectrum antibiotics, immunosuppressive therapies, and invasive medical procedures in hospital settings, which create selective pressures that promote the emergence and persistence of multidrug-resistant pathogens.

Importantly, the role of HGT in facilitating the spread of ARGs is significant. This process allows bacteria to acquire new genetic traits, including those that confer antibiotic resistance, from other microorganisms. Hospital wastewater, not municipal wastewater, affects HGT, increasing the proportion of recipients harboring resistance [90]. Bacteriophages serve as reservoirs for ARGs, including *bla*, *mecA*, *mcr-1*, and *vanA*. These ARGs can be reintroduced into bacterial cells through HGT [91], thereby facilitating the dissemination of ARGs within environmental contexts. This transmission mechanism also plays a role in the environmental prevalence of *blaNDM-1*. The study’s authors have shown that *blaNDM-1* is encoded by self-transmissible plasmids, as well as several other plasmids, present in hospital wastewater [33]. In the present study, hospital wastewater contained a high abundance of MGEs, such as phages (two times greater) and plasmid-related functions (3.8 times greater), than domestic sewage wastewater, revealing a risk of HGT in hospital wastewater [92].

A WWTP is a conventional type of sewage treatment. Hospital wastewater is usually discharged into WWTPs and co-treated with municipal wastewater. The WHO recommends that hospital wastewater be treated on-site, subsequently discharged into WWTPs, and treated with other wastewater. CAS, disinfection processes, chlorination, and UV disinfection or ozonation are often used in traditional WWTPs. ARB and ARG concentrations decreased significantly after treatment with WWTPs in most studies. However, ARB and ARG in hospital wastewater cannot be eliminated and can spread resistance through WWTPs. In our review, new technologies, such as membrane bioreactor treatment, ozonation, and E-peroxone-SBR, resulted in the positive elimination of ARGs. More advanced treatment technologies will be developed to remove emerging contaminants from wastewater. Moreover, we should also consider the important role of pressure selection and horizontal transfer in the treatment of hospital wastewater. These methods may induce the expression of error-prone DNA polymerases and HGT of ARGs. More studies should be conducted to treat ARGs in hospital wastewater or other places.

COVID-19 has caused a global storm, subsequently inducing bacterial infections and hindering antibiotic stewardship [93,94,95]. The extensive application of antibiotics has led to a significant rise in the levels of residual antibiotics being released into WWTPs [96,97]. Additionally, the environmental presence of biocides and single-use plastics has markedly escalated. Antibiotics interact synergistically to enhance the proliferation of ARB and ARGs. It is imperative to continue monitoring the progression of AMR in the context of post-COVID-19 conditions (long COVID), with a concentrated emphasis on low- to middle-income countries, where the challenges posed by ARBs and ARGs are more severe and where treatment resources are inadequate.

In summary, hospital wastewater serves as a primary source of ARB and ARGs. WWTPs also function as reservoirs and environmental contributors to antibiotic resistance, acting as hotspots for HGT, and facilitating the dissemination of ARB and ARG in the environment. Further research is necessary to explore the prevalence of resistance genes and to evaluate various effective treatment processes.

## 6. Conclusions

This review highlights that HWW is a significant source of clinically important ARB and ARGs, with much higher concentrations than municipal, community, or rural wastewater. Key findings include the following:

*Carbapenem resistance*: HWW is dominated by carbapenemase genes (*blaNDM-1* and *blaKPC*) far exceeding non-hospital levels (e.g., *blaNDM-1*: 71% prevalence in hospital settings in contrast to 12% in community settings).

*MRSA*: The *mecA* gene is common in HWW (3.26 × 10^7^ copies/100 mL) and retirement home sewers but not found in municipal wastewater.

*VRE*: The *vanA* gene shows a prevalence of 7.95 × 10^1^ copies/100 mL in HWW, with hospitals contributing more than community sources.

*ESBLs*: Genes like *blaCTX-M* are more abundant in HWW (7.96 log copies/mL) compared to urban wastewater (6.86 log copies/mL).

*Other resistance*: *mcr*-1 is present in HWW (2.23 × 10^4^ copies/100 mL) but is not detected in retirement home settings.

Current wastewater treatment methods vary in effectiveness. Conventional methods like chlorination and activated sludge reduce ARGs, albeit inconsistently; for example, chlorination decreased LARGs but had negative removal rates for *blaTEM-1* and *tetB*. Advanced technologies such as membrane bioreactors and ozonation perform better, achieving over 3 log reductions for *blaSHV* and *ermB*. The E-peroxone-SBR system also effectively reduced *qnrS* and *aac(6′)-Ib-cr*. However, challenges remain, including incomplete ARG removal, high costs, toxic byproducts, and bacterial regrowth after UV treatment.

The COVID-19 pandemic has intensified the challenges associated with antimicrobial resistance, as evidenced by an 88.5% prevalence of ARGs in COVID-19 isolation hospitals. This situation is primarily attributed to the extensive use of antibiotics, affecting between 33% and 95% of patients, and the horizontal gene transfer of carbapenem-resistant genes.

Based on the status of ARB and ARGs in hospital wastewater, a multipronged strategy is essential to address these challenges.

(1) Publicity and education: The WHO established a global campaign called “The World AMR Awareness Week (WAAW)” to raise awareness and understanding of AMR, encouraging global action. However, much more attention is paid to patients than to wastewater in hospitals, so it is critical to strengthen public awareness and education on the importance of hospital sewage treatment;
(2) The life-cycle management of antibiotics: Guidelines to reduce non-essential antibiotic use in clinical settings should be strengthened and stricter pretreatment regulations for HWW discharge into the environment should be enforced. Meanwhile, we also need to establish monitoring systems, tracking ARG dynamic distribution in real time;
(3) Innovative treatment technologies: First, we should upgrade existing facilities and build on-site wastewater treatment equipment. Second, we should also make more efforts to develop innovative treatment technologies, with an emphasis on cost-efficiency and compatibility with existing infrastructure;
(4) Integrated emergency equipment development: It is essential to formulate secure medical equipment to enable the rapid and efficient treatment of HWW during a Public Health Emergency of International Concern (PHEIC), such as the COVID-19 pandemic.

In summary, urgent multidisciplinary efforts should be made to mitigate ARB and ARGs’ dissemination and public health impacts in the future.

## Figures and Tables

**Table 1:** CRE comparison between HWW and wastewater from other places.

Study	Numbers of Samples	ARB	Hospitals	Other Places	ARG	Hospitals	Other Places
[27]	7 (H), 20 (RH), 32 (HSG)	-	-	-	Copies/100 mL (*blaKPC3*)	1.64 × 10^2^	-
					Copies/100 mL (*blaNDM-1*)	3.13 × 10^3^	4.28 × 10^3^ (RH) NA (HSG)
					Copies/100 mL (*blaOXA48*, *blaCTX-M32*, *blaCTX-M15*, *blaCMY-2*)	3.95 × 10^6^	2.59 × 10^6^ (RH) 1.23 × 10^6^ (HSG)
[28]	49 (H), 45 (WWTP)	Number of isolates to *Klebsiella* apecies	49	45	Number of resistance genes (median number)	9	4 (WWTP, Serving the hospital and local community, *p* < 0.001)
[29]	21 (HWW), 21 (UWW)	Anaerobic human gut bacteria and Enterobactertiales (%)	(H > U, *p* < 0.05)		HWW/UWW (ARGs or MGEs)	3–161 fold (*p* < 0.004)	
[30]	218 (clinic), 196 (mix), 200 (city)	Citrobacter species (%)	H > M, *p* < 0.05		log copies/mL (*blaNDM*, *blaCTX-M-15*)	clinic mixed with city > city (*p* < 0.01, *p* < 0.05)	
		Enterobacter/Pseudomonas species (%)	H > M, *p* < 0.001		log copies/mL *blaVIM*	clinic > city (*p* < 0.01)	
[31]	-	Bacterial species (number)	25	18 (UR)	ARGs (number)	47	22 (UR)
[25]	9 (H), 16 (RS)	XDR isolates (%)	26/475 (5.47%)	0/568 (0%)	Detected *blaNDM* (no. of genes)	70	0
					Detected *blaKPC*	1	0
					Detected *blaOXA51*	4	0
		Carbapenemase genes (%)	134/475 (28.21%)	8/568 (1.41%)	Detected *blaGIM*	5	0
					Detected *blaOXA48*	45	0
					Detected *blaIMI*	0	4
					Detected *blaVIM*	36	4
[32]	20 (H), 20 (WWTP)	CPE isolates (number)	7	0	Detected *blaNDM*	1	0
[33]	72 (H), 41 (COM)	NDM^+^/all samples (%)	51/72 (71%)	5/41 (12%) (*p* < 0.001)	*blaNDM-1*^+^/all samples (%)	51/72 (71%)	5/41 (12%) (*p* < 0.001)
[34]	72, 20	CRE logCFU/mL	7.25	4.09	log copies/mL *blaNDM*	13.2	5.42
[26]	77 (H), 36 (CMO)	-	*-*	*-*	*blaNDM-1*^+^/all samples (%)	2/77 (3%)	1/36 (3%)
					*blaKPC*^+^/all samples (%)	37/77 (48%)	16/36 (44%)
[35]	-	-	-	-	log (*blaTEM* copies/mL)	Hospital effluent > upstream, downstream (*p* < 0.05)	

Hospital (H), Retirement home (RH), Housing (HSG), Wastewater treatment plant (WWTP), Hospital wastewater (HWW), Urban wastewater (UWW), Upstream river water (UR), Rural system (RS), Community (CMO).

**Table 2:** Comparison of MRSA prevalence between hospital wastewater and wastewater from other places.

Study	Number of Samples	ARB	Hospitals	Other Places	ARG	Hospitals	Other Places
[27]	7 (H) 20 (RH)	-	-	-	Copies/100 mL (*mecA*)	3.26 × 10^−7^	2.55 × 10^−7^~8.19 × 10^−5^
[36]	6 (H), 10 (M)				Detected *mecA*	positive	negative
[29]	21 (HWW), 21 (UWW)	Anaerobic human gut bacteria and Enterobactertiales (%)	(H > U, *p* < 0.05)		HWW/UWW (ARGs or MGEs)	3–161 fold (*p* < 0.004)	
[26]	53 (H), 50 (CMO)	MRSA^+^/all samples (%)	5/53 (9%)	0/50 (0%)			

Hospital (H), Retirement home (RH), Municipal (M), Hospital wastewater (HWW), Urban wastewater (UWW), Community (CMO).

**Table 3:** VRE comparison between HWW and wastewater from other places.

Study	Number of Samples	ARB	Hospitals	Other Places	ARG	Hospitals	Other Places
[27]	7 (H) 20 (RH) 32 (HSG)	-	-	-	Copies/100 mL (*vanA*)	7.95 × 10^1^	-
[37]	4 (H), 44 (CMO)	-	-	-	Detected *vanC*	3	3
[26]	54 (H), 55 (CMO)	VRE^+^/all samples (%)	18/54 (33%)	6/55(11%)	-	-	-
[38]	7 (H), 42 (CMO)	-	-	-	prevalence of *vanA*	Significantly higher H >> CMO	
[39]	16 (H), 42 (U)	VRE^+^/all samples (%)	14/16 (87%)	20/30 (67%)	Detected *vanA*	14	20
[40]	26 (H), 21 (U)	Colony-forming units/100 mL VRE	1.3 × 10^5^	5.7 × 10^5^	Detected *vanA*	2	3
					Detected *vanB*	2	6
[41]	14 (H), 12 (R)	VRE^+^/all samples (%)	11/14 (78.6%)	0/12 (0%)	-	-	-
[36]	6 (H), 10 (M)	VRE (%)	25%	12.5%	Detected *vanA*	positive	positive
[42]	14 (H), 35 (Raw Water)	Prevalence of VRE8 resistance (%)	43% in Sweden	57% in Sweden	-	-	-
	23 (H), 49 (Raw Water)		30% in Spain	98% in Spain	-	-	-
	22 (H), 21 (Raw Water)		18% in UK	67% in UK	-	-	-
[43]	14 (H) 37 (SW)	VRE^+^/all samples (%)	5/14 (36%)	1/37 (3%)	Detected *vanA*	1	1
					Detected *vanB*	4	0

Hospital (H), Retirement home (RH), Housing (HSG), Community (CMO), Urban (U), River (R), Municipal (M), Surface water (SW).

**Table 4:** ESBL comparison between hospital wastewater and wastewater from other places.

Study	Number of Samples	ARB	Hospitals	Other Places	ARG	Hospitals	Other Places
[44]	17 (H), 5 (RW)	ESBL production in *E. coli* (%, *Klebsiella pneumoniae*)	35%, 50%	15%, 0%	Detected *blaCTX*	1	1
					Detected *blaTEM*	4	1
					Detected *blaSHV*	3	1
[45]	2644 (H) 2525 (CMO) 2693 (U)	ESBL (%)	11.5%	6.9% (CMO) 3.7% (U)	-	-	-
[34]	72, 20	ESBL (logCFU/mL)	7.29	3.97	log copies/mL *blaTEM*	7.08	7.36
					log copies/mL *blaOXA*	6.35	5.31
					log copies/mL *blaCTX*	7.96	6.86
[46]	6 (H), 17 (U)	ESBL isolates (%)	14.9%	2.9%	Detected *blaCTX-M*	14	12
		ESBL (%)	13.6%	2.3%	Detected *blaTEM*	18	0
[47]	45 (H), 60 (U)	ESBL *E. coli* (CFU/mL)	27 × 10^3^	0.75 × 10^3^ (*p* < 0.001)	Detected *blaSHV*	2	5
		ESBL *E. coli*/total *E. coli* (%)	7.8%	0.1%	Detected *blaTEM*	7	4
[48]	21 (H), 21 (M)	ESBL isolates (%)	37.1%	17.7%	Detected *blaCTX-M*	41	7
					Detected *blaTEM*	60	11
					Detected *blaSHV*	6	3
					Detected *blaOXA*	11	0
[49]	17 (H), 27 (M)	ESBL/isolates (%)	5/106 (4.7%)	1/36 (2.8%)	Detected *blaCTX-M*	5	1
					Detected *blaSHV*	1	0
					Detected *blaTEM*	1	0

Hospital (H), Recreational water (RW), Community (CMO), Urban (U), Municipal (M).

**Table 5:** Comparison of other resistance genes between hospital wastewater and wastewater from other places.

Study	Number of Samples	ARB	Hospitals	Other Places	ARG	Hospitals	Other Places
[27]	7 (H) 20 (RH) 32 (HSG)	-	-	-	Copies/100 mL (*mcr-1*)	2.23 × 10^−6^	NA (due to absence of *mcr-1* in RH)
[50]	15 (H), 15 (M)	-	-	-	mcr-1^+^/all samples	6/15	11/15 (municipal, *p* = 0.139, but higher levels in H, *p* = 0.007)
					mcr-3/4/5	H < Municipal (*p* < 0.001)	
					cfr (A)^+^/all samples	15/15	N.D.
					optrA^+^/all samples	13/15	N.D.
					*sul4*	15/15	15/15 (municipal)
					gar	15/15	15/15 (municipal)
[34]	72, 20	-	-	-	log copies/mL *Int1*	10.4	8.44
[51]	Total 66 (H, M)	Gentamicin- resistant bacteria (CFU/100 mL)	1.84 × 10^7^	6.29 × 10^6^	Detected *aac(3)-1*	2/3	3/3
		Chloramphenicol	2.84 × 10^7^	3.73 × 10^7^	Detected *cmlA1*	2/3	2/3
		Ceftazidim	7.36 × 10^7^	3.73 × 10^7^	Detected *ctx-m-32*	2/3	0/3
[36]	6 (H), 10 (M)	-	-	-	Detected *amp*C	positive	positive
[30]	218 (clinic), 196 (mix), 200 (city)	Citrobacter species (%)	higher, *p* < 0.05		log copies/mL *bla_NDM_*	Mixed (clinic and city) > city (*p* < 0.01)	
					log copies/mL *bla_CTX-M-15_*	Mixed (clinic and city) > city (*p* < 0.05)	
		Enterobacter/Pseudomonasspecies (%)	higher, *p* < 0.001		log copies/mL *bla_VIM_*	clinic > city (*p* < 0.01)	
					log copies/mL *sul1*	Mixed (clinic and city) > city (*p* < 0.05)	
					log copies/mL *mcr-1*	Mixed (clinic and city) > city	
[42]	105 (H), 59 (Raw Water)	Prevalence of ERE8 resistance (%)	86% in Sweden	26% in Sweden	-	-	-
			83% in Spain	100% in Spain	-	-	-

Hospital (H), Retirement home (RH), Housing (HSG), Municipal (M), Recreational water (RW).

## Data Availability

No new data were created or analyzed in this study. Data sharing is not applicable to this article.
